# Genome evolutionary dynamics followed by diversifying selection explains the complexity of the *Sesamum indicum* genome

**DOI:** 10.1186/s12864-017-3599-4

**Published:** 2017-03-24

**Authors:** Jingyin Yu, Linhai Wang, Hui Guo, Boshou Liao, Graham King, Xiurong Zhang

**Affiliations:** 1 The Key Laboratory of Biology and Genetic Improvement of Oil Crops, Ministry of Agriculture, Oil Crops Research Institute, the Chinese Academy of Agricultural Sciences, Wuhan, 430062 China; 2 Plant Genome Mapping Laboratory, the University of Georgia, Athens, GA 30605 USA; 3 Southern Cross Plant Science, Southern Cross University, PO Box 157, Lismore, NSW 2480 Australia; 4 The Key Laboratory of Crop Genetic Improvement, Huazhong Agricultural University, Wuhan, China

**Keywords:** Genome evolution, Dynamics, Whole genome duplication, Tandem duplication, Function divergence

## Abstract

**Background:**

Whole genome duplication (WGD) and tandem duplication (TD) provide two critical sources of raw genetic material for genome complexity and evolutionary novelty. Little is known about the complexity of the *Sesamum indicum* genome after it diverged from a common ancestor with the paleodiploid *Vitis vinifera* and further experienced WGD and TD events.

**Results:**

Here, we analyzed the functional divergence of different classes of inter- and intra-genome gene pairs from ancestral events to uncover multiple-layers of evolutionary dynamics acting during the process of forming the modern *S. indicum* genome. Comprehensive inter-genome analyses revealed that 60% and 70% of syntenic orthologous gene pairs were retained among the two subgenomes in *S. indicum* compared to *V. vinifera*, although there was no evidence of significant differences under selection pressure. For the intra-genomic analyses, 5,932 duplicated gene pairs experienced fractionation, with the remaining 1,236 duplicated gene pairs having undergone functional divergence under diversifying selection. Analysis of the TD events indicated that 2,945 paralogous gene pairs, from 1,089 tandem arrays of 2–16 genes, experienced functional divergence under diversifying selection. Sequence diversification of different classes of gene pairs revealed that most of TD events occurred after the WGD event, with others following the ancestral gene order indicating ancient TD events at some time prior to the WGD event. Our comparison-of-function analyses for different classes of gene pairs indicated that the WGD and TD evolutionary events were both responsible for introducing genes that enabled exploration of novel and complementary functionalities, whilst maintaining individual plant ruggedness.

**Conclusions:**

In this study, we first investigated functional divergence of different classes of gene pairs to characterize the dynamic processes associated with each evolutionary event in *S. indicum*. The data demonstrated massive and distinct functional divergence among different classes of gene pairs, and provided a genome-scale view of gene function diversification explaining the complexity of the *S. indicum* genome. We hope this provides a biological model to study the mechanism of plant species formation, particularly in the context of the evolutionary history of flowering plants, and offers novel insights for the study of angiosperm genomes.

**Electronic supplementary material:**

The online version of this article (doi:10.1186/s12864-017-3599-4) contains supplementary material, which is available to authorized users.

## Background

**Fig. 1:**
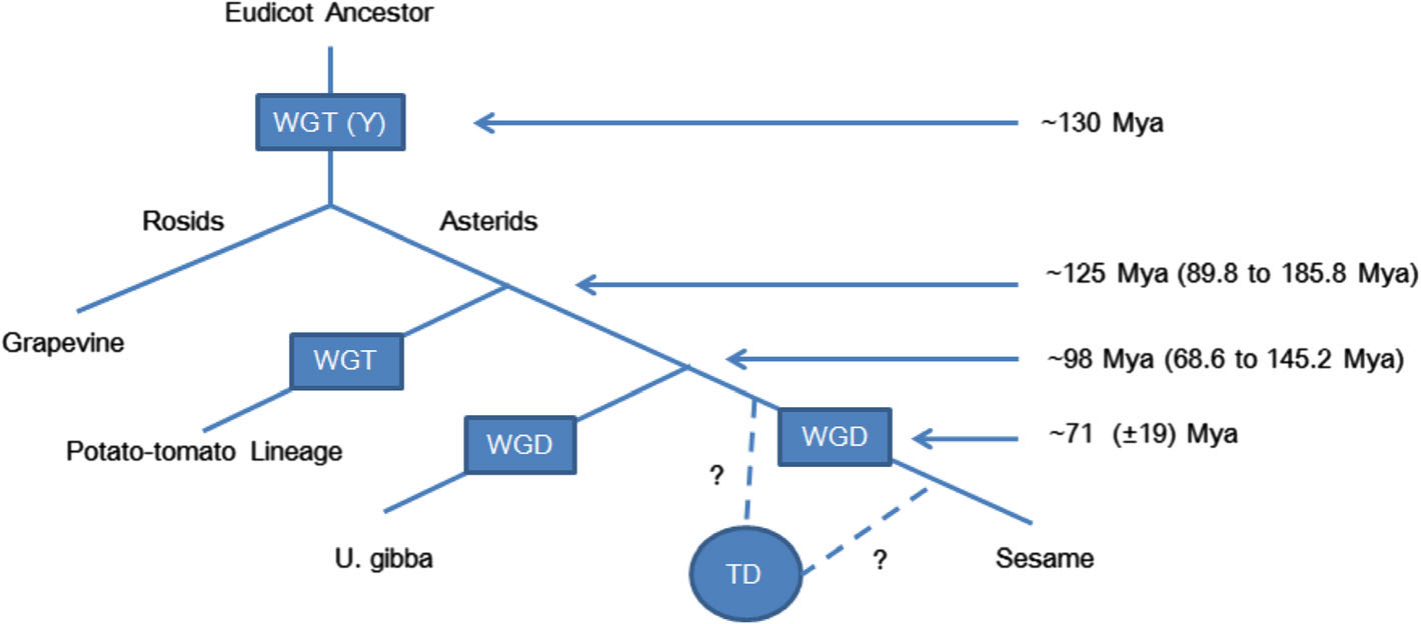
Ancestral polyploid events and corresponding timeline within the asterids lineage. Rectangles represents whole genome duplication events and ovals tandem duplication events. WGT: whole genome triplication. WGD: whole genome duplication. TD: tandem duplication. Question mark (?) represents undetermined occurrence time of tandem duplication event

Whole genome duplication (WGD) has been an important driving force in accelerating angiosperm diversification, and is recognized as the primary source of novel genomic material contributing to genome complexity and evolutionary novelty [1]. The prevalence of WGD in flowering plants has been detected by analyzing the evidence of ancestrally inherited gene duplicates [2, 3]. Phylogenetic analyses of gene duplicates has uncovered two ancient WGD events that occurred at the root of the seed plants (ζ), and at the base of the angiosperms (Ɛ) prior to the divergence of monocots and eudicots. These events were estimated to occur around 319 ± 3 and 192 ± 2 million years ago (Mya) [4]. Within the eudicot lineage, phylogenetic analyses indicate an ancient whole genome triplication (WGT) event (Ƴ) that predated the split of the Asterid and Rosid lineages approximately 130 Mya [2, 5–7]. Based on evolutionary relationships between plant species in the asterid clade, the *S. indicum* genome has been estimated to have diverged from the *Solanum* lineage approximately 125 Mya (89.8 to 185.8 Mya), and from *U. gibba* approximately 98 million years ago (68.6 to 145.2 Mya). By comparing the distribution of synonymous codon (Ks) mutations for duplicated genes from WGD events among *S. indicum*, *U. gibba* and *Solanum* lineages, the most recent WGD in the lineage leading to *S. indicum* was estimated to have occurred approximately 71 (±19) Mya, possibly at the same evolutionary stage and in parallel with the WGT event within the *Solanum* lineage [8] (Fig. 1).

There are many genome-sequenced plant species in the rosid clade, but relatively few in the asterid clade. In the rosid clade, *Vitis vinifera* was the fourth species for which the complete genome sequence was established in flowering plants. After comparison with its close relatives, *V. vinifera* was considered as a true diploid, which had not undergone recent genome duplication [9]. So, *V. vinifera* was thought to contain ancient genomic loci or ancestral gene orders, which could be used to enable the discovery of ancestral traits and genomic features of flowering plants. In the asterid clade, prior to the release of the *Sesamum indicum* (sesame*,* Asteraceae) draft genome [8], several genomes were publicly available, including *Solanum tuberosum* (potato), *Solanum lycopersicon* (tomato), *Utricularia gibba* (floating bladderwort) and *Mimulus guttatus* (monkey flower), which had experienced WGD or WGT events or near-doubling of chromosome numbers within their genomes [6, 7, 10–12]. Therefore, *V. vinifera* represents a paleodiploid species that is close to plant species in the asterid clade, and which experienced the older eudicot genome triplication event (γ) [6–8, 10, 13]. As a result, the paleodiploid *V. vinifera* in the rosid clade has maintained a complement of single-copy genes or single-copy syntenic regions at a whole-genome scale compared to other taxa within the asterid clade. Previous comparison of the two modern genomes, *S. indicum* and *V. vinifera*, has led to identification of two non-overlapping subgenomes in the *S. indicum* genome, which provides a rich source of genomic data to study orthologous genes between *V. vinifera* and *S. indicum*, as well as duplicated genes in *S. indicum* [8].

The WGD event contributed duplicated genes leading to the increase of gene dosage in *S. indicum*. Previous study indicated that duplicated genes mainly originate as a result of four different processes, that include ectopic recombination, replication slippage, retro-transposition and WGD [14]. Following duplication, these genes may experience different evolutionary fates under diversifying selection pressures, including conserved function, sub-functionalization [15, 16], neo-functionalization [17, 18] and loss [19]. Followed by diversifying selection in an evolutionary process, duplicated genes from the three WGD events in the *A. thaliana* lineage provided functional divergence and indicated sub- and neo-functionalization, which have been evaluated by protein-protein interaction in modern *A. thaliana* populations [20]. Moreover, the relative gene expression of paralogous genes across tissues demonstrates that 98% of duplicate pairs have sub-functionalized in a tissue-wise manner following WGD events [21]. Tandem duplication (TD) is a ubiquitous phenomenon in flowering plants, which can also bring about the increase of gene dosage [22, 23]. Compared to other duplication events, TDs occur more frequently and focus on smaller scale duplication within the genome [24, 25]. TD events are prevalent in many flowering plants and are a characteristic feature of many gene families related to key traits or phenotypes, including the genes coding for nucleotide binding site (NBS), cytochrome P450s and receptor-like kinases [26–28]. The tandem duplicated genes generated by TD events have experienced functional divergence under diversifying selection. From expression difference analysis of the NBS-encoding gene family in *Brassica rapa* and *B. oleracea*, paralogous genes from tandem arrays contributed more towards functional divergence than orthologous genes between *B. rapa* and *B. oleracea* over their evolutionary history [26].

Both WGD or TD events can contribute to anincrease in gene dosage, which may enhance the biological function of duplicated genes. However, duplicated genes from WGD event or paralogous genes from TD events may subsequently display functional divergence, which was not explained by the gene-dosage balance hypothesis [29]. Several questions therefore arise: How the gene-dosage balance hypothesis influence gene evolution in *S. indicum*? How is the function of the gene changed in the evolutionary history of the *S. indicum* genome? What isthe complexity of the *S. indicum* genome after it diverged from a common ancestor with *V. vinifera* (species divergence event), and experienced WGD and TD events?

In this study, we first compared two *S. indicum* subgenomes and the *V. vinifera* genomes to obtain syntenic orthologous gene pairs. Secondly, we inferred duplicated gene pairs in the *S. indicum* subgenomes attributable to the WGD event. Thirdly, we identified pairs of genes based on every possible combination from a tandem array to constitute two-gene paralogous gene pairs in a corresponding tandem array within the *S. indicum* genome. Using different classes of gene pairs from the *S. indicum* specific ancient evolutionary events, we investigated the functional divergence of different classes of gene pairs by employing InterPro annotation to trace the evolutionary dynamic process of *S. indicum* genome followed by diversifying selection. From comparison of functional divergence for different classes of gene pairs, we characterized the dynamics associated with each evolutionary event to determine the complexity of the *S. indicum* genome. The data demonstrate massive and distinct functional divergence among different gene pairs, and provide a genome-scale view of gene function diversification which is able to be traced to ancient evolutionary events. We propose that these insights into the dynamics of *S. indicum* genome evolution serve as an important model for studying the evolutionary biology of flowering plants.

## Results

### Influence of whole genome duplication on the *S. indicum* genome

**Fig. 2:**
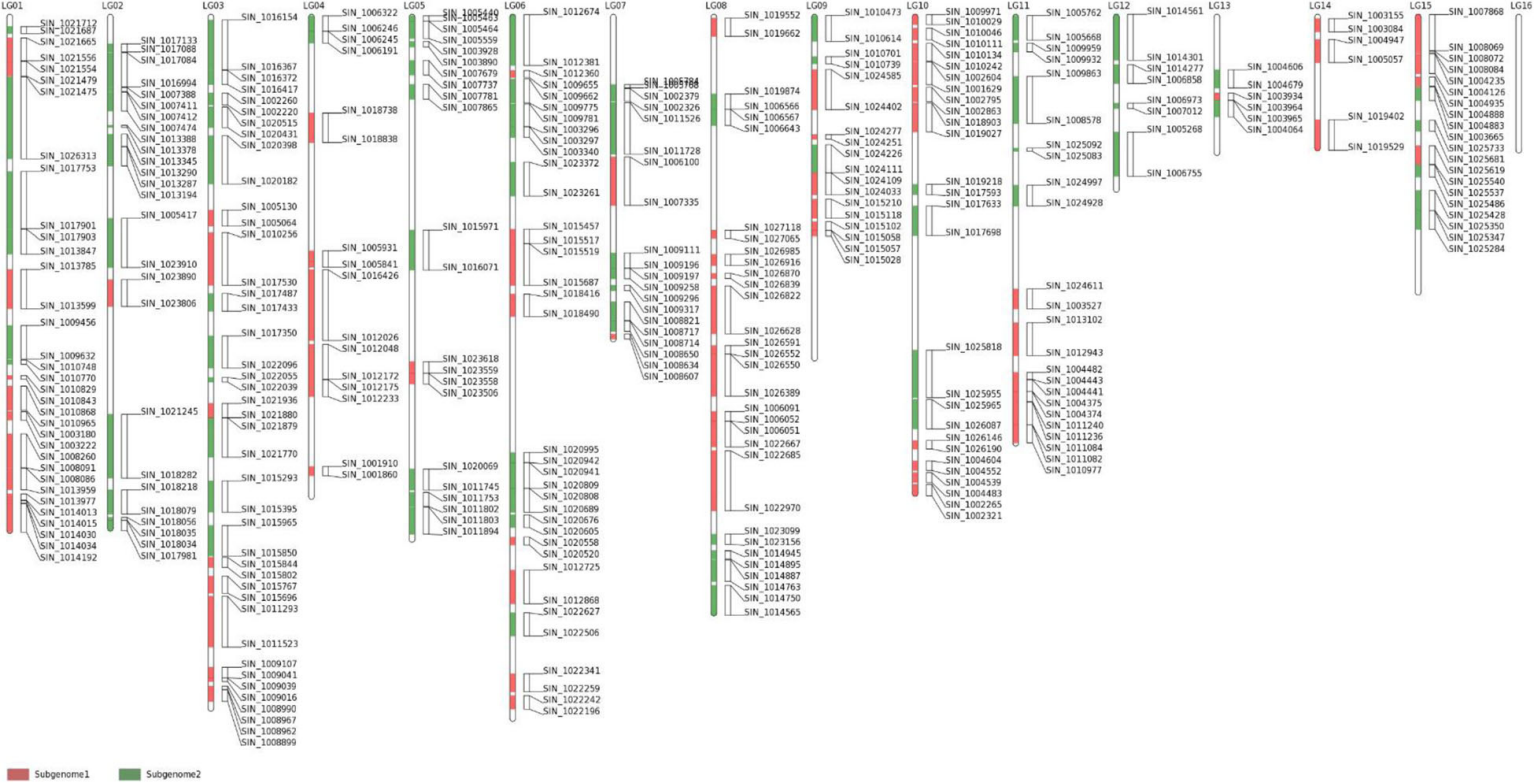
Alignement of the *S. indicum* subgenomes compared to the *V. vinifera* genome. The bar represents the pseudomolecule chromosomes. *Red bar*: *S. indicum* ‘Subgenome1’; *green bar*: ‘Subgenome2’

*S. indicum* has experienced a WGD event approximately 71 (±19) Mya, which resulted in two subgenomes (Subgenome1 and Subgenome2) compared to the *V. vinifera* genome [8]. Employing *S. indicum* and *V. vinifera* genomes, we used blastp to reconstruct orthologous gene pairs between the two species with an E-value threshold of 1e-20 [30]. We then employed the MCscanX program to identify orthologous genomic regions with the parameters (e = 1e-20, u = 1 and s = 15) between *S. indicum* and *V. vinifera* genomes [31]. After manual curation, Subgenome1 covered approximately 57.22 Mb (7,450 genes) represented by 82 syntenic blocks in common with the *V. vinifera* genome. Meanwhile, Subgenome2 covered approximately 68.66 Mb (7,958 genes) represented by 87 syntenic blocks in common with the *V. vinifera* genome (Additional file 1: Table S1). Together, the two subgenomes represented 45.9% of the assembled *S. indicum* genome, which included 56.7% of the 27,148 genes currently annotated within *S. indicum* genome (Table 1). Each chromosome pseudomolecule apart from ‘LG16’ contained a few syntenic regions, indicating that the *S. indicum* genome has experienced chromosome fragmentation and reassortment following the WGD event. The number of syntenic regions identified within the pseudo-chromosomes varied from 3 (LG14) to 21 (LG03). The longest syntenic region of 2.93 Mb on ‘LG01’ was associated with Subgenome1, as was the shortest syntenic region of 63 Kb on ‘LG15’ (Fig. 2).

**Table 1:** Summary statistics of syntenic regions on *S. indicum* subgenomes

Categories	No. of syntenic blocks	Genomic length (Mb)	Gene numbers
Subgenome1	82	57.22	7450
Subgenome2	87	68.66	7958
Total syntenic blocks:	169		
Total genomic length:		125.88	
Total gene numbers:			15,408
Genome length:		274	
Genome gene numbers:			27,148
Percentage (Total */Genome *)		45.94%	56.76%

### Functional divergence of syntenic orthologous gene pairs

Based on the syntenic relationships, we identified 5,932 *V. vinifera* genes that had orthologous genes located within the *S. indicum* subgenomes. This comparison involved 3,656 and 3,512 syntenic orthologous genes in Subgenome1 and Subgenome2, respectively. InterPro annotation enabled us to annotate these syntenic orthologous genes with functional descriptions [32]. We allocated each of the syntenic orthologous gene pairs to one of three classes, depending on their functional-divergence status: (A) conserved function, with shared identical InterPro entries, (B) sub-functionalization, with shared partially identical InterPro entries, and (C) neo-functionalization, with completely different InterPro entries. The A, B and C functional divergence classes were used as evidence of collinearity between orthologous gene pairs in the *S. indicum* genome and corresponding ancient genomic loci in the *V. vinifera* genome.

Of the 3,656 syntenic orthologous gene pairs shared between Subgenome1 and the *V. vinifera* genome, 2,681 (73.3%) shared the same InterPro entries, indicating that these genes retained conserved functions with the ancient genomic loci present in *V. vinifera*. A total of 471 (12.9%) orthologous gene pairs retained partially identical InterPro entries, indicating they had undergone sub-functionalization following the *S. indicum* split from a common ancestor with the paleodiploid *V. vinifera*.154 (4.2%) orthologous gene pairs had unrelated InterPro entries, suggesting that these genes had undergone neo-functionalization in *S. indicum* (Additional file 2: Table S2). Of the 3,512 syntenic orthologous gene pairs between Subgenome2 and the *V. vinifera* genome, 3,117 were represented by InterPro entries, with 2,537 (81.4% of those annotated) sharing identical InterPro entries, indicating that these retained the same function as the corresponding ancient genes in the *V. vinifera* genome. A total of 449 (12.8% of those annotated) shared partial InterPro entries and 132 (4.2% of those annotated) had distinct InterPro entries, suggesting that the function of these two classes of *S. indicum* genes had undergone sub-functionalization and neo-functionalization compared to orthologues in *V. vinifera* (Table 2, Additional file 3: Table S3).

**Table 2:** Comparison of different classes of gene pairs between *V. vinifera* genome and *S. indicum* subgenomes

Categories	Total No. of Gene Pairs	No. of Gene Pairs with no Annotation	No. of Gene Pairs with Conserved Function	No. of Gene Pairs with Neofunctionalization	No. of Gene Pairs with Subfunctionalization
*V. vinifera* and Subgenome1	3,656	350	2,681	154	471
*V. vinifera* and Subgenome2	3,512	395	2,537	132	449

### Selection pressure on syntenic orthologous gene pairs

For coding sequences, the strength of selection pressure is measured by the ratio of the rates of nonsynonymous substitution over synonymous substitutions (Ka/Ks) [33, 34]. We calculated Ka/Ks of *S. indicum* and *V. vinifera* syntenic orthologous gene pairs to determine whether they had experienced different selective pressures during the process of functional divergence. After filtering gene pairs with low sequence similarity, we found that the remaining 3,306 syntenic orthologous gene pairs, representing 90.42% of the total 3,656 syntenic orthologous gene pairs, from each of the A, B, C classes associated with Subgenome1 had a low mean Ka/Ks ratio (0.142, median value: 0.126). This result indicates that they had experienced purifying selection. The A class orthologous gene pairs with identical InterPro entries had the lowest mean Ka/Ks ratio (0.131, median value: 0.118), suggesting relatively strongpurifying selection, whereas the C class orthologous gene pairs had the highest mean Ka/Ks ratio (0.203, median value: 0.181), indicating that they had experienced weaker purifying selection. The Ka/Ks ratio (0.140, median value: 0.121) for the B class gene pairs was intermediate, although overall there were significant differences between each class (Mann-Whitney *U* test, P_A:B_ = 0.03916 < 0.05; P_A:C_ = 2.2e-16 < 0.05; P_B:C_ = 2.852e-12 < 0.05).

In comparison, we found that overall, 3,116 syntenic orthologous gene pairs from the A, B, C classes associated with Subgenome2 had a mean Ka/Ks ratio of 0.121 (median value: 0.128), which is lower than the mean Ka/Ks ratio observed between Subgenome1 of the *S. indicum* genome and the *V. vinifera* genome. However, this difference was not statistically significant (Mann-Whitney *U* test, P = 0.3371 > 0.05). A similar pattern of Ka/Ks ratios was found (A: 0.131, median value: 0.119; B: 0.142, median value: 0.13 and C: 0.179, median value: 0.166), and a similar inference of purifying selection for any two classes (Mann-Whitney *U* test, P_A:B_ = 0.003246 < 0.05; P_B:C_ = 1.016e-08 < 0.05; P_A:C_ = 0.0001182 < 0.05) with the consensus evolutionary pattern under diversifying selection among different classes of functional divergence.

### Fractionation of duplicated gene pairs

**Fig. 3:**
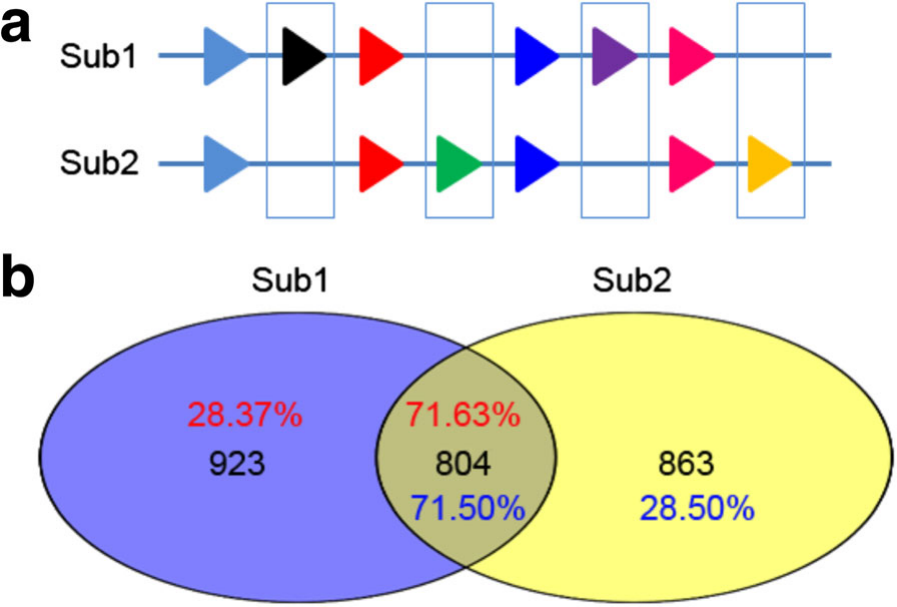
Fractionation of duplicated gene pairs in *S. indicum* subgenomes. **a**. Fractionation of duplicated gene pairs on syntenic regions within Subgenome1 and Subgenome2. Line represents genomic regions collinear between the *S. indicum* subgenomes. Identical colored triangless represent syntenic gene pairs. Boxes represent fractionation of duplicated gene pairs. **b**. Function divergence indicated by InterPro entries. Venn diagram indicates relationships between genes and InterPro entries of asymmetric retained genes in duplicated gene pairs. The integer indicates number of InterPro entries of asymmetric retained genes in duplicated gene pairs. The percentage represents the proportion of gene numbers with InterPro entries to total genes in Subgenome1 or Subgenome2

Using the *V. vinifera* genome to represent the reference ancient genome, we extracted two syntenic subgenomes from *S. indicum* in order to detect the evolutionary fate of duplicated genes following the WGD event. Duplicated gene pairs located on two syntenic subgenomes in the *S. indicum* genome will tend to fractionate following a WGD event (Fig. 3a). Based on loss and retention of duplicated gene pairs, we found that 4,696 duplicated gene pairs (79.16%) experienced fractionation, with 2,420 gene pairs retained in Subgenome1 and 2,276 in Subgenome2. There are 1,236 gene pairs co-retained in the two subgenomes in *S. indicum* (Table 3). We next focused on the fractionation of duplicated gene pairs in *S. indicum*, as represented by colored boxes in Fig. 3a. The functional analysis of duplicated genes in *S. indicum* provides a novel approach to detect asymmetric evolution within duplicated subgenomes. In order to compare the functions of single-copy genes retained in the different *S. indicum* subgenomes, we employed the InterPro entries to describe the gene function based on the characteristics of conserved domains. This enabled us to identify 923 (28.37% of the genes in Subgenome1) InterPro functional entries for single-copy genes within Subgenome1 and 863 (28.5% of the genes in Subgenome2) from Subgenome2. Interestingly, we found 804 InterPro functional entries shared between the two subgenomes. These results suggest that the two *S. indicum* subgenomes still retained many genes with identical function, although they had experienced fractionation of duplicated gene pairs. The specific InterPro entries found in Subgenome1 were enriched for gene families or conserved functional domains of the GNAT domain, Ubiquitin carboxyl-terminal hydrolases family 2, Glycoside hydrolase, family 5, actin-binding, cofilin/tropomyosin type, peptidase S54, rhomboid, peptidase M20, cation-transporting P-type ATPase, N-terminal, cation-transporting P-type ATPase, C-terminal, peptidase S54, and the rhomboid domain. The specific InterPro entries related to Subgenome2 were enriched for gene families or conserved functional domains of phosphatidylinositol 3-/4-kinases and catalytic domain which participated in biological process of defense response and response to biotic stimulus. The most prevalent common InterPro entries between the two subgenomes included gene families or conserved functional domains of Protein kinase domain, Serine/threonine- /dual specificity protein kinase, catalytic domain, Tyrosine-protein kinase, catalytic domain and pentatricopeptide repeats, which participate in the biological processes of the protein phosphorylation signal transduction system, as well as carbohydrate biosynthesis and metabolism (Fig. 3b).

**Table 3:** Statistics of fractionation and retention of duplicated genes from *S. indicum* subgenomes

Categories	Total No. of Gene Pairs	No. of retained genes	No. of co-retained genes	No. of fractionated or specific retained genes
Subgenome1	5,932	3,656	1,236	2,681
Subgenome2		3,512		2,537

### Functional divergence of duplicated gene pairs following the whole genome duplication event

Functional divergence of duplicated gene pairs followed by WGD is an important dynamic process for plant genome evolution. WGD events increase gene dosage and provide the opportunity for subsequent functional divergence. Out of 1,236 duplicated gene pairs between the two subgenomes, 110 duplicated gene pairs were not annotated by InterPro entries. After removing these unannotated duplicated gene pairs, we found 74.11% (916 duplicated gene pairs) for the A class shared identical InterPro entries, suggesting that they had maintained common functions following the WGD. We found that these included conserved functional domains for protein kinase domain, Serine/threonine-/dual specificity protein kinase, catalytic domain, tyrosine-protein kinase, catalytic domain, SANT/Myb domain, AAA+ ATPase domain, Myc-type, basic helix-loop-helix (bHLH) domain, Myb domain and homeobox domain, mainly enriched into gene families of protein kinase and transcription factors. For the B class, 130 duplicated gene pairs shared incomplete InterPro entries, indicating that these duplicated gene pairs had undergone partial functional divergence under selection pressure with sub-functionalization in each subgenome. For the 238 InterPro entries referred to Subgenome1, 45 InterPro entries were subgenome1-specific and 193 InterPro entries overlapped with Subgenome2. About 59 of all 252 InterPro entries were subgenome2-specific InterPro entries and the remaining 193 InterPro entries overlapped with Subgenome1. The sub-functionalized duplicated gene pairs annotated with the overlapped InterPro entries between Subgenome1 and Subgenome2 were classified into the gene families of protein kinase and transcription factors, suggesting that although the duplicated gene pairs have undergone sub-functionalization, the important functions of duplicated gene pairs were also maintained and enriched for the same gene families which played an important role in the growth and development processes in *S. indicum*. For the C class, we detected 80 duplicated gene pairs that shared completely different InterPro entries among the subgenomes. The members of duplicated gene pairs in Subgenome1 were annotated by 54 different InterPro entries, and the members of duplicated gene pairs in Subgenome2 were annotated by 80 different InterPro entries. Of these, 16 common entries were mainly associated with the conserved domains or motifs of zinc finger, RING-type 3 (IPR001841), SANT/Myb domain (IPR001005), pentatricopeptide repeat (IPR002885) and Myb domain (IPR017930) involved in the molecular function of zinc ion binding and chromatin binding (Additional file 4: Table S4).

The duplicated genes with conserved function or sub-functionalization in different subgenomes were mainly enriched into conserved domains or motifs of protein kinases and transcription factors, which represented a larger proportion of all duplicated gene pairs. The neo-functionalized duplicated gene pairs experienced severe functional divergence, although these genes still InterPro entries in common which mainly focused on the conserved domains or motifs of zinc finger and transcription factors. These results suggested that WGD events had primarily brought about an increase in protein kinases and transcription factors involved in biological processes of signal transduction system, protein phosphorylation and signal transduction, carbohydrate biosynthesis and metabolism, as well as transcriptional regulation [35].

### Selection underlying the functional divergence of duplicated genes

The analysis of functional annotation for duplicated gene pairs with InterPro entries suggested that about 16.99% of 1,236 duplicated gene pairs have diverged in function after WGD including the duplicated gene pairs in classes of sub-functionalization and neo-functionalization. To investigate the selection pressures of duplicated gene pairs within *S. indicum*, we analyzed the Ka/Ks ratios of 1,236 duplicated gene pairs having different types of functional divergence as annotated by InterPro entries. Results showed a low mean Ka/Ks ratio (0.193, median value: 0.177) indicating that the duplicated genes had experienced purifying selection. The duplicated gene pairs of the A class of conserved function have the lowest mean Ka/Ks ratio (0.174, median value: 0.163), indicating these genes had undergone the strongest purifying selection compared to the gene pairs of the B and C classes. The mean Ka/Ks ratio of duplicated gene pairs in the B class was 0.212 (median value: 0.191), which was significantly greater than that from the A class. The analysis reveals the B class duplicated genes experienced weaker purifying selection than that of A class, and the mean Ka/Ks ratios for duplicated gene pairs were found to differ significantly between the A and B classes (Mann-Whitney *U* test, P_A:B_ = 0.001043 < 0.05). The mean Ka/Ks ratio of C class duplicated gene pairs (0.27, median value: 0.252) was significantly greater than that of B class duplicated gene pairs, which indicated that the duplicated gene pairs of C class had been subject to the weakest purifying selection amongst different classes of duplicated gene pairs within syntenic regions in *S. indicum*. The mean Ks in the A class (0.852, median value: 0.69) was similar to that of the B class (0.845, median value: 0.696), although the average Ks in the C class (1.6, median value: 0.949) was significantly greater than that of A and B classes, suggesting that the C class duplicated genes accumulated more synonymous mutations and showed greater sequence divergence. Another possible interpretation of elevated Ks in class C genes where these genes have substantially lower overall similarity, is that the sequence alignments for these genes were more error prone, which would artificially elevate synonymous substitutions. The mean Ka in the C class (0.452, median value: 0.227) was also significantly higher than that of the A (0.141, median value: 0.119) and B (0.176, median value: 0.142) classes, indicating that the A and B class duplicated gene pairs may have accumulated fewer single base substitutions and experienced weaker purifying selection, thus making the function of duplicated gene pairs more conserved.

### Influence of tandem duplication events in the *S. indicum* genome

**Fig. 4:**
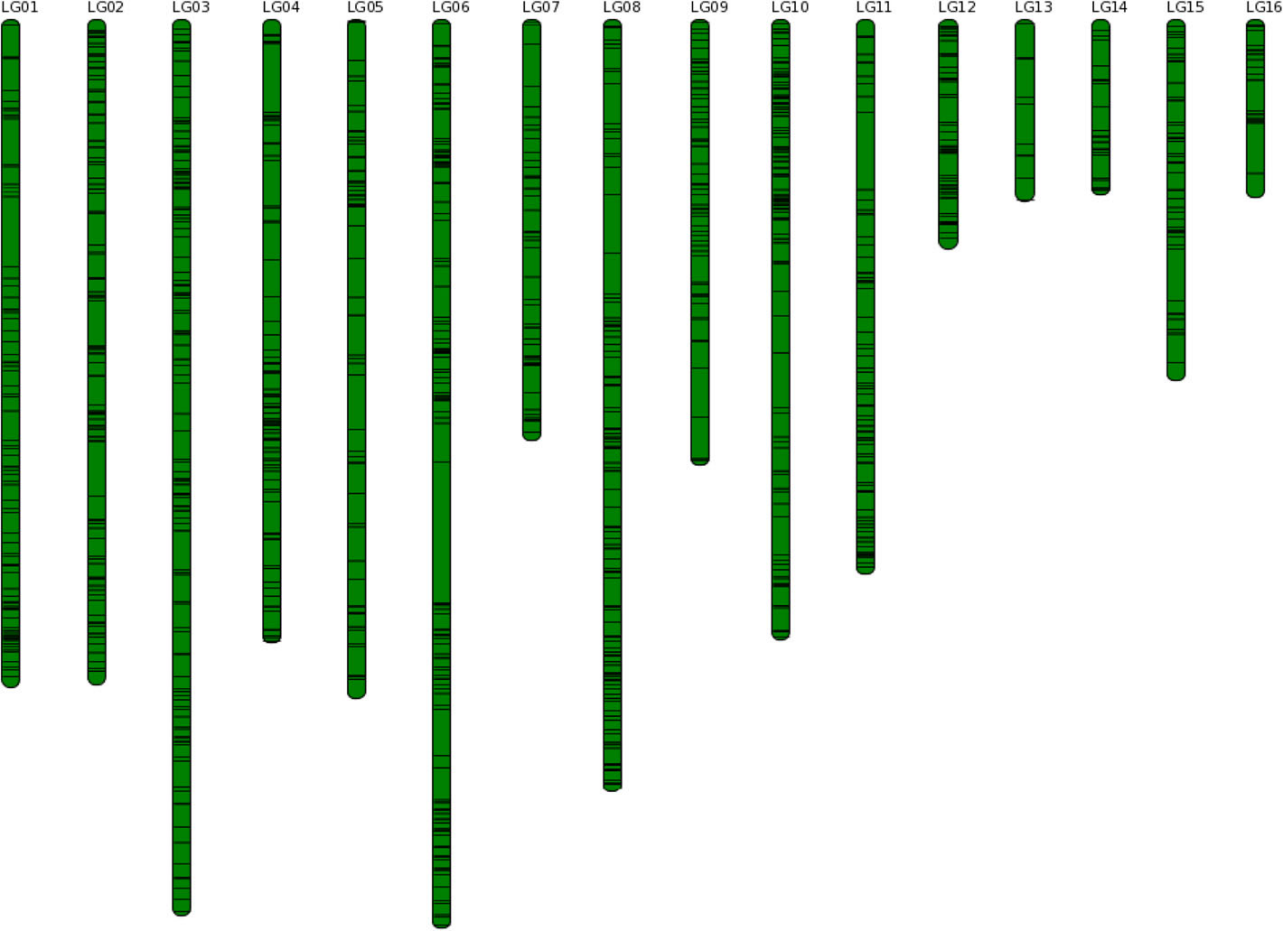
Distribution of tandem duplicated genes in *S. indicum* genome. *Green bars* represent pseudo-molecular chromosomes. *Black horizontal line* on *green bars* represents tandem duplicated genes in *S. indicum* genome

Tandem duplication events will lead not only to the expansion of gene families, but also an increase of gene dosage in the form of tandem arrays [36]. Tandem duplicated genes in the *S. indicum* genome have previously been reported and were available in the PTGBase database [37]. We used this set and curated them based on the characteristics of conserved domains or motifs of gene families. This provided a set of 2,745 tandem duplicated genes distributed in 1,089 tandem arrays of 2–16 genes for further analysis (Additional file 5: Table S5). From the *S. indicum* genome, 2,570 of the tandem duplicated genes representing 94% of total tandem duplicated genes, were distributed in 1,008 tandem arrays, and anchored on the 16 linkage groups (LG), with an uneven distribution. The highest proportion was anchored on the LG06, with 290 tandem duplicated genes distributed in 118 tandem arrays of 2–9 genes. The LG06 contained 2,745 protein-coding genes and tandem duplicated genes represented 10.56% of total protein-coding genes in LG06. In contrast, the LG13 contained 21 tandem duplicated genes generated by 9 tandem arrays of 2–4 genes. The LG13 contained 522 protein-coding genes and tandem duplicated genes represented 4.02% of total protein-coding genes in LG13. The largest tandem array consisted of 16 genes on LG12, and these genes were involved in the molecular function of oxidoreductase activity and flavin adenine dinucleotide binding [35] (Fig. 4).

### Function divergence between the members of tandem array

For each tandem array, we selected two genes based on every possible combination to constitute paralogous gene pairs to investigate functional divergence. For example, one tandem array has three genes (a, b and c), which will generate three paralogous gene pairs (a-b, a-c and b-c). Finally, we obtained 2,945 paralogous gene pairs among all tandem arrays. Based on the annotation by InterPro entries, 197 of the paralogous gene pairs (6.7%) were not represented by InterPro entries. We therefore used the annotation of InterPro entries to determine the functional divergence of 2,748 paralogous gene pairs in tandem arrays, of which 2,308 (78.4%) sharing identical InterPro entries were classified into the A class of conserved function. These were mainly recognized as the members of gene families or conserved domains of Auxin-induced protein, ARG7, Cytochrome P450 and Cytochrome P450, E-class, group I. 425 (14.4%) shared partially identical InterPro entries and were allocated as sub-functionalized between members of paralogous gene pairs, and were grouped into the gene families or conserved domains of Protein kinase domain, Serine/threonine-/dual specificity protein kinase, catalytic domain and Tyrosine-protein kinase, catalytic domain. Only 15 gene pairs were recognized as the gene pairs of complete functional divergence and were also grouped into the gene families of protein kinases. This analysis indicates that the majority of tandem duplicated genes has a conserved function. Irrespective of whether the paralogous genes belonged to the members of gene pairs with sub-functionalization or neo-functionalization, the paralogous gene pairs of functional divergence represented a smaller proportion in all paralogous gene pairs of tandem arrays. This suggests that most of tandem duplicated genes in *S. indicum* display a bias towards conserved function, suggesting the tandem duplicated genes were subject to weaker selection pressure (Additional file 6: Table S6).

### Gene functional differences between duplicated and tandem duplicated genes

**Fig. 5:**
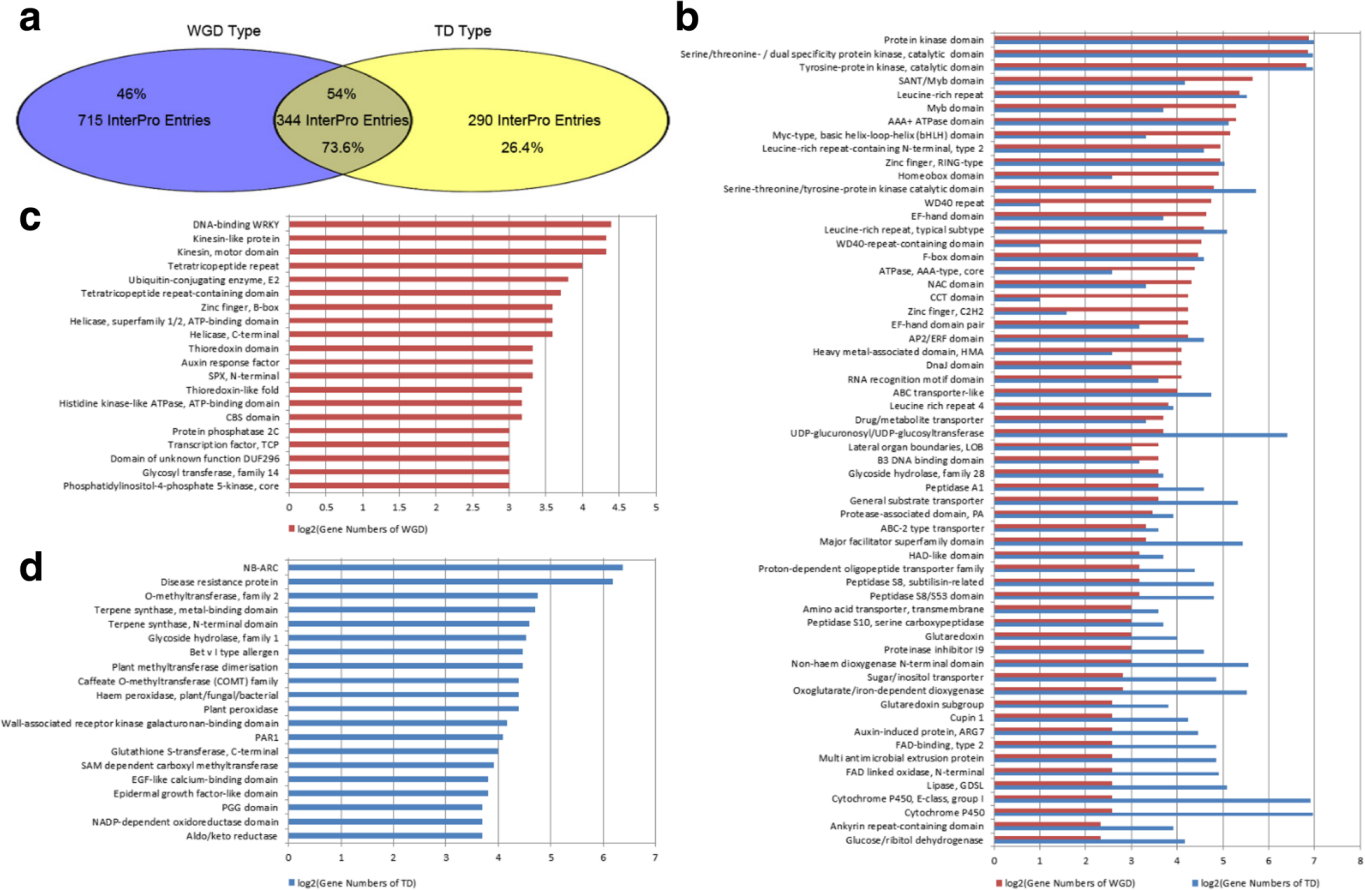
Functional differences of gene pairs from WGD and TD events. **a**. Functional differences of gene pairs from WGD and TD events by InterPro entries. Venn diagram shows the numbers of genes and InterPro entries of different gene pairs from WGD and TD events. The integer indicates the number of InterPro entries of different gene pairs. The percentage numbers represent the proportion of gene numbers in different gene pairs with InterPro entries to total genes from WGD or TD event. **b**. Comparison of InterPro entries of different gene pairs from WGD and TD events. *Red bars* represent the numbers after Log2 normalization of duplicated gene pairs from WGD event. *Blue bars* represent the numbers after log2 normalization of paralogous gene pairs from TD event. **c**. The specific InterPro entries of duplicated gene pairs from WGD event. *Red bars* represent the numbers after log2 normalization of duplicated gene pairs. **d**. The specific InterPro entries of paralogous gene pairs from TD event. *Blue bars* represent the numbers after Log2 normalization of paralogous gene pairs

WGD and TD events provide abundant genomic materials and bring more opportunities for species to adapt to changing environments under selection pressure. Since these two events occurred during different stages of evolutionary history, we propose that the InterPro annotation is able to detect gene functional differences between the two events. Approximately 1,059 InterPro entries were used to annotate 2,472 duplicated genes from WGD event in *S. indicum*, and 634 InterPro entries for 2,745 tandem duplicated genes from TD events in *S. indicum*, providing evidence that the WGD event introduced greater gene complexity with distinct functional ingredients compared to the TD events. From the comparative analysis of annotation between WGD and TD events, we obtained 344 overlapping InterPro entries between duplicated and tandem duplicated genes. The remaining 715 InterPro entries were used to annotate 46% of all duplicated genes, which consisted of specific InterPro entries for annotation of duplicated genes. Removing the overlapping InterPro entries, the remaining 290 InterPro entries were specific for tandem duplicated genes, which were used to annotate 26.4% of all tandem duplicated genes (Fig. 5a). Gene numbers were compared following Log2 normalization between duplicated and tandem duplicated genes, which were annotated by the overlapping InterPro entries. For function comparison, the members of gene families for protein kinase represented the largest proportion of all tandemly duplicated genes, consistent with that in duplicated genes, although the gene number was different between the two datasets. Members of the Cytochrome P450 gene family were over-represented within tandem duplicated genes, whereas transcription factors represented a larger proportion of duplicated genes (Fig. 5b). Members of the transcription factor WRKY and Kinesin gene families were classified according to specific InterPro entries for duplicated genes (Fig. 5c), with disease resistance recognition genes (R genes) enriched within the specific InterPro entries within tandem duplicated genes (Fig. 5d). Thus the different evolutionary events affecting the *S. indicum* genome have given rise to a different complement of genes with distinct functional ingredients. From this analysis, it appeared that the resulting gene composition generated by WGD and TD events differed as a result of selection. Some families with relatively high retention frequencies for TD events have relatively low retention frequencies for the WGD event, and *vice versa* [38].

### Sequence diversification of different classes of gene pairs from evolutionary events

**Fig. 6:**
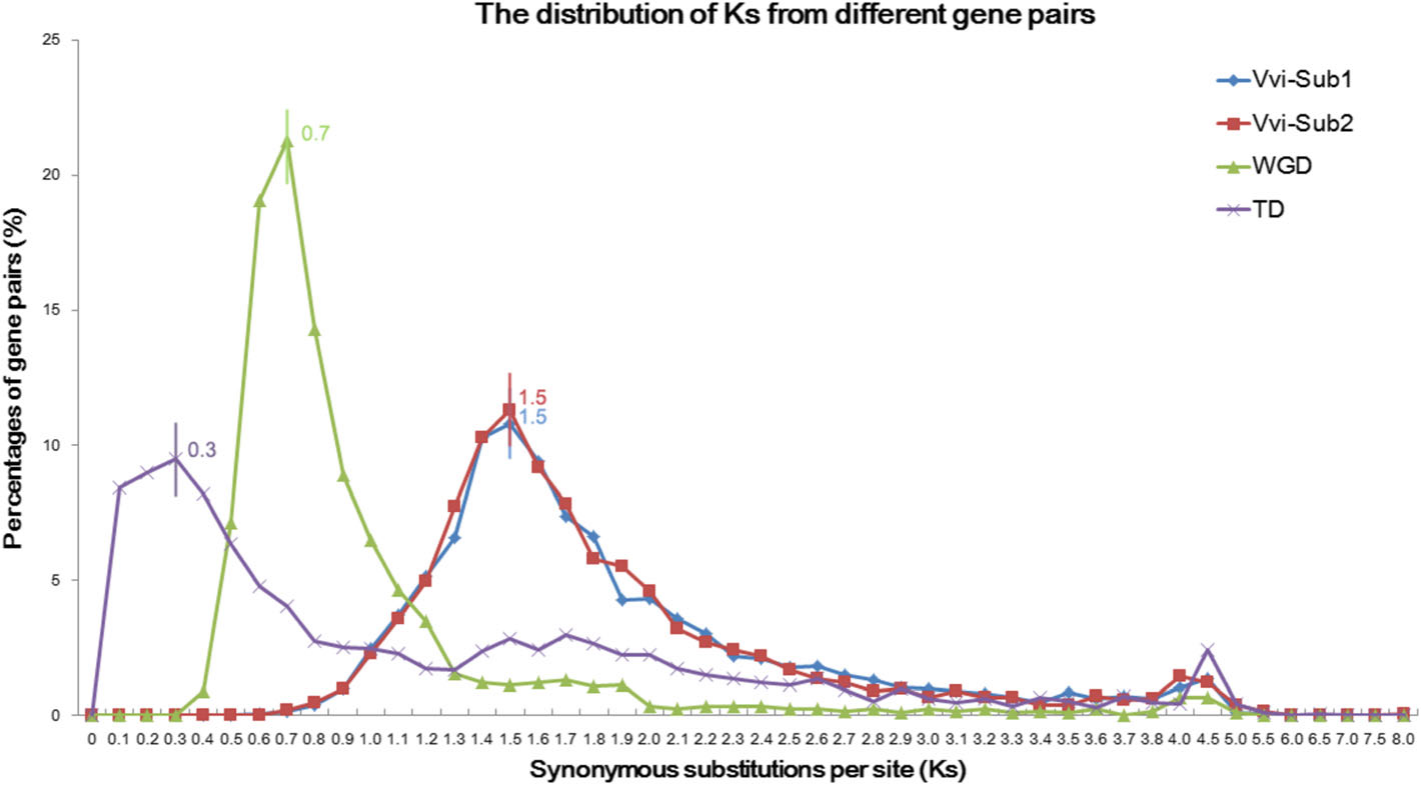
Distribution of synonymous substitutions per site (Ks) in gene pairs from different evolutionary events. Vvi-Sub1, *V. vinifera* compared to the Subgenome1 in *S. indicum*. Vvi-Sub2, *V. vinifera* compared to the Subgenome2 in *S. indicum*. WGD, whole genome duplication event; TD, tandem duplication event. Ks values are shown on each peak

In order to detect sequence diversification of different classes of gene pairs arising from specific evolutionary events, we extracted different classes of gene pairs from different evolutionary processes, including 3,649 syntenic orthologous gene pairs from Subgenome1 compared to the *V. vinifera*, 3,510 syntenic orthologous gene pairs from Subgenome2 compared to the *V. vinifera*, 1,230 duplicated gene pairs from the WGD event and 2,913 paralogous gene pairs from the TD events, to obtain their synonymous substitution rates (Ks) values. From comparison of Ks values of different classes of gene pairs, we observed nearly parallel and identical peaks (1.5) in the Ks distributions for the syntenic orthologous gene pairs between *V. vinifera* and *S. indicum* subgenomes. The maximum Ks for duplicated gene pairs from WGD event was 0.7, which fitted the ranges of 0.5 − 1 Ks from a more recent WGD event of *S. indicum*, indicating that the most common recent ancestor of *S. indicum* and *V. vinifera* was a diploid relative to *V. vinifera* and that the lineages which gave rise to the two subgenomes of the modern sesame genome diverged from each other well after the split of the *S. indicum* and *V. vinifera* lineages. The maximum Ks for paralogous gene pairs from tandem arrays was 0.3, which was the lowest of different classes of gene pairs from the other evolutionary events, indicating that the most of TD events may have occurred more recently than the WGD event, and also later than the *S. indicum* split from a common ancestor with *V. vinifera* (Fig. 6).

### Dating of tandem duplication events in *S. indicum*

The analysis of sequence divergence of different classes of gene pairs indicates that most of the TD events represented the most recent events in the evolutionary history of *S. indicum*, and likely occurred after the WGD event. In order to date the evolution of tandem duplicated genes, we combined the different classes of gene pairs from the WGD and TD events. 126 tandem duplicated genes distributed in 63 two-gene tandem arrays were located on the *S. indicum* subgenomes, which had 118 syntenic orthologous genes in *V. vinifera*, suggesting that these genes were located on syntenic blocks in the subgenomes compared to *V. vinifera* genome, and may be inherited from their ancestral gene orders (Additional file 7: Table S7). There was no evidence for the remaining 2,619 tandem duplicated genes being associated with the ancient genomic loci, indicating that these tandem duplicated genes might be generated after the WGD event. With the WGD event recognized as a reference point, tandem duplicated genes can then be divided into two classes: 126 tandem duplicated genes which were generated before the WGD event, and 2,619 after. Of the first set 72 tandem duplicated genes are distributed within 36 two-gene tandem arrays and are located within Subgenome1, with the remainder (54) distributed on 27 two-gene tandem arrays in Subgenome2. From these results, we concluded that the TD events had not occurred at a particular evolutionary stage but had been a continuous process over a long historical period, which is consistent with the description of the *Brassica* genus [39].

### Evolutionary patterns of certain gene families followed by whole genome duplication and tandem duplication events

Through the analysis of gene functional differences between duplicated and tandem duplicated genes in *S. indicum*, some gene families were affected more by theWGD event, but others were more affected by TD events. In order to detect the evolutionary patterns of gene families following the WGD and TD events, certain gene families containing conserved domains of DNA-binding WRKY, NB-RAC and Cytochrome P450 were chosen to investigate the consequences of each event. The DNA-binding WRKY gene family is one of the largest families of transcriptional regulators in plants and contributes integral parts of signaling pathways modulating many plant processes [40]. Based on the InterPro annotation, 72 WRKY genes were detected in the *S. indicum* genome. It appears that 21 (29.2%) of these were generated by the WGD event, and none by TD events. The NB-ARC (NBS-encoding) gene family is a major class of disease resistance recognition genes (R genes), and play an important role in defense against pests and pathogens, thus improving the adaptability of plants to biotic stress [41]. Approximately 171 NBS-encoding genes were detected by the InterPro annotation in the *S. indicum* genome, of which 83 (48.5%) were generated by TD and none by WGD. Cytochrome P450 monooxygenases constitute a large superfamily of heme-thiolate proteins prevalent in prokaryotes and eukaryotes [42, 43], and involved in biosynthesis of fatty acids, structural polymers (lignins), pigments (anthocyanins), accessory pigments (carotenoids), defense-related compounds (some phytoalexins), and UV protectants (flavonoids and sinapoyl esters) [44]. According to the InterPro annotation, 307 cytochrome P450 genes were extracted, representing 1.13% of the gene complement within the *S. indicum* genome, of which six were generated by WGD and 126 generated by TD, indicating a more significant influence of TD than WGD event for this gene class in *S. indicum* (Table 4).

**Table 4:** Comparison of the members of WRKY, NBS-encoding and Cytochrome P450 gene family after WGD and TD events

Gene families	Total No. of gene families	Generated by WGD event	Generated by TD event
WRKY	72	21	0
NBS-encoding	171	0	83
Cytochrome P450	307	6	126

## Discussion

### Functional divergence by diversifying selection

The study of functional-divergence for different classes of gene pairs has been explored in the context of the three ancestral WGD events leading to the contemporary *Arabidopsis* genome. Different proportions of duplicated gene pairs from these sequential WGD events have indicated functional divergence using the number of identified protein-protein interactions as a proxy. Differences between duplicated gene pairs based on Gene Ontology annotation have reinforced this evidence of functional divergence from protein-protein interactions, and has been interpreted as indicative of adaptation to different cellular components [20]. Comparison of functional divergence between the two *S. indicum* subgenomes compared to the *V. vinifera* genome, indicates that 73.3% of Subgenome1 and 72.2% of Subgenome2 have retained a conserved function between members of gene pairs, with the remainder displaying evidence of sub-functionalization or neo-functionalization. Functional analysis of diverged gene pairs indicates enrichment for different functional classes. The analysis of selection pressures indicated that the syntenic orthologous gene pairs can be assigned to those with conserved function, sub-functionalization and neo-functionalization, resulting from different selection pressures. *S. indicum* has experienced distinct genomic events at different evolutionary stages, with each resulting in extensive changes in composition of gene pairs. Moreover, it appears that some duplicated gene pairs subsequently emerged with a distinct evolutionary fate under diversifying selection, including sub-functionalization and neo-functionalization. Taken together, these results suggest that these classes of genomic event led to the introduction of extensive novel genomic materials resulting in different classes of gene pairs, with evidence of adaptive evolution under diversifying selection. This appears to have provided novel opportunities for species adaptation to changing environments.

### Gene functional compensation followed by whole genome duplication and tandem duplication events

Based on the functional differences 1,059 InterPro entries were used to annotate duplicated genes and 634 to annotate tandemly duplicated genes in *S. indicum*. Of these, 344 had shared InterPro entries*,* with the remainder allocated to WGD-specific and TD-specific events. This analysis indicated that such gene pairs were mainly grouped into gene families involved in plant development and growth, but the TD-specific InterPro entries were mainly classified into gene families related to environmental influence. Based on genome-wide comparative analysis of NBS-encoding genes between *Brassica* species and *Arabidopsis*, Yu et al. (2014), demonstrated that the TD events led to an increase in gene dosage of NBS-encoding genes resulting in gene amplification, which may have some advantages for plant parasite defense [26]. The TD events giving rise to expansion of the NBS-encoding gene family is also likely to have benefited the resistance of *S. indicum* to the diseases and pests, and improve the adaptation to a changing environment. The WGD and TD events have brought specific genes with different functional features to the *S. indicum* genome, which appear to have been essential genomic ingredients for plant growth and development. Where the WGD event has not brought sufficient functional components to meet the need for survival or increased fitness, the TD events have been a valuable mechanism to generate additional genomic ingredients to maintain plant fitness. We infer that this may be due to a critical mechanism for functional compensation in plant evolutionary history, and the mutual compensation of genes, through synergies with each other, jointly maintained the ruggedness of *S. indicum*.

### Gene evolutionary dynamics arising from evolutionary events

The ancestral *S. indicum* genome has diverged from a common ancestor with the ancestral *V. vinifera* and inherited evolutionary evidence of ancestral gene orders. Subsequently, the ancestral *S. indicum* genome has experienced a WGD event around 71 (±19) Mya, which introduced extensive additional genomic materials leading to genome-wide chromosome fragmentation and rearrangement. TD events, which increase gene dosage and contribute to the expansion of gene families, have occurred over a long historical evolutionary period, although most of them have occurred mainly after the WGD event. The WGD and TD events increased gene dosage and improved the corresponding gene function, which will increase the likelihood of plant survival in changing environments. This can be explained by the gene-dosage balance hypothesis [29]. Subsequently, some duplicated genes or tandem duplicated genes experienced sub-functionalization or neo-functionalization under diversifying selection, which did not fit the gene-dosage balance hypothesis. So, the gene-dosage balance hypothesis might influence certain periods in the evolutionary history of *S. indicum* genome. Following each evolutionary event, functional components of the *S. indicum* genome have undergone subsequent gene functional divergence, and meanwhile also generated novel functional components. The WGD and TD events have independently supplied novel genomic materials, each complementing the other in terms of functional components, and both contributing to the additional functional features and ruggedness of the species.

## Conclusions

The availability of the *S. indicum* genome sequence provides an opportunity to investigate the characterization of *S. indicum* genome, and to compare with genomic analogues in its closely relatives through a comparative genomics approach. By tracing the evolutionary history of *S. indicum* it appears that WGD and TD events occurred after the divergence of the predecessors of *S. indicum* and *V. vinifera* from a common ancestor. These evvents have also provided an extensive genomic resource to investigate the complexity of the *S. indicum* genome. According to syntenic relationship between *S. indicum* and *V. vinifera*, 60% and 70% of syntenic orthologous gene pairs were retained among Subgenome1 and Subgenome2 in *S. indicum* compared to *V. vinifera*. Based on selection pressure analysis, there was no evidence of significant differences between different subgenomes in *S. indicum* compared to *V. vinifera*. For the intra-genomic analyses, 5,932 duplicated gene pairs were retained 3,656 and 3,512 single-copy genes in Subgenome1 and Subgenome2 compared to *V. vinifera* respectively, which meant that duplicated gene pairs in *S. indicum* have experienced fractionation. The co-retained 1,236 duplicated gene pairs in different subgenomes in *S. indicum* have undergone functional divergence under diversifying selection. From comparison of WGD and TD events, most of tandem duplicated genes were generated after the WGD, with others following the ancestral gene order indicating ancient tandem duplication at some time prior to the WGD. Our comparison of function analyses revealed that the WGD and TD evolutionary events were both responsible for introducing genes that enabled exploration of novel and complementary functionalities. Importantly, the comparison of gene families related to certain traits or phenotypes and their further exploitation may help us to uncover the intriguing evolutionary process of special traits or phenotypes in *S. indicum*, which can explore the phenotypic diversity due to the complexity of *S. indicum* genome. We hope this provides a valuable biological model to study the mechanism of plant species formation, particularly in the context of the evolutionary history of flowering plants, and offers a novel insight for the study of angiosperm genomes.

## Methods

### Data resources

*S. indicum* and *V. vinifera* genomic and annotation data were downloaded from the Sinbase (http://ocri-genomics.org/Sinbase/) [45] and Genoscope (http://www.genoscope.cns.fr) [13], respectively. The putative tandem duplicated genes in *S. indicum* genome were downloaded from the PTGBase (http://ocri-genomics.org/PTGBase/) [37].

### InterPro annotation Analysis

In order to provide functional analysis of protein sequences by classifying them into families and predict the presence of domains and important sites, the functional domains or conserved sites classification for a gene was determined by the InterPro database [46]. All records were derived from member databases of the InterPro consortium by using predictive models, known as signatures. Gene function divergence in the members of gene pairs is defined by sharing partially identical or complete differences InterPro entries between different classes of gene pairs.

### Gene Ontology annotation

Gene Ontology was employed to determine the functional enrichment analysis for the members of different classes of gene pairs by predicting the presence of conserved domains or important sites [35].

### Calculation of Ka, Ks and Ka/Ks Values

Protein sequences of different classes of gene pairs were aligned using ClustalW [47]. Coding sequence alignments of different classes of gene pairs were guided by protein sequence alignment using PAL2NAL [48]. Nonsynonymous substitutions per sites (Ka) and synonymous substitutions per sites (Ks) values were calculated using the yn00 program in the PAML package [33].

## Additional files

Additional file 1: Table S1. The syntenic gene pairs in *S. indicum* compared to *V. vinifera*. (XLSX 176 kb)

Additional file 2: Table S2. Functional divergence of different classes of syntenic gene pairs in *S. indicum* Subgenome1 compared to *V. vinifera* genome. (XLSX 428 kb)

Additional file 3: Table S3. Functional divergence of different classes of syntenic gene pairs in *S. indicum* Subgenome2 compared to *V. vinifera* genome. (XLSX 419 kb)

Additional file 4: Table S4. Functional divergence of different classes of duplicated gene pairs between the two subgenomes in *S. indicum*. (XLSX 172 kb)

Additional file 5: Table S5. Tandem duplicated genes in *S. indicum*. (XLSX 78 kb)

Additional file 6: Table S6. Functional divergence of different classes of the members in tandem array. (XLSX 219 kb)

Additional file 7: Table S7. The ancient tandem duplicated genes located on subgenomes in *S. indicum*. (XLSX 11 kb)
